# Dynamic Changes in Aroma Compounds during Processing of Flat Black Tea: Combined GC-MS with Proteomic Analysis

**DOI:** 10.3390/foods13203243

**Published:** 2024-10-12

**Authors:** Cun Ao, Xiaojun Niu, Daliang Shi, Xuxia Zheng, Jizhong Yu, Yingbin Zhang

**Affiliations:** 1 Tea Research Institute, Hangzhou Academy of Agricultural Science, Hangzhou 310024, China; aocun123@163.com (C.A.); xiaojunwords@126.com (X.N.); sdl7698@126.com (D.S.); xxzheng43@163.com (X.Z.); 2 Tea Research Institute, Chinese Academy of Agricultural Sciences, Hangzhou 310008, China

**Keywords:** black tea, aroma compound, dynamic change, processing technology

## Abstract

Flat black tea (FBT) has been innovatively developed to alleviate homogenisation competition, but the dynamic changes in aroma components during the process remain unclear. This study employed HS-SPME-GC-MS to analyse the aroma components of tea samples from various processing stages of FBT, and to make a comparative assessment with conventional strip-like Congou black tea (SBT). Additionally, a proteomic analysis was conducted on fresh leaves, withered leaves, and frozen–thawed leaves. Significant changes were observed in the aroma components and proteins during the processing. The results of the multivariate and odour activity value analysis demonstrated that the principal aroma components present during the processing of FBT were linalool, (E)-2-hexen-1-al, methyl salicylate, geraniol, hexanal, benzeneacetaldehyde, (Z)-3-hexenyl butyrate, dimethyl sulphide, 2-methylbutanal, 2-ethylfuran, nonanal, nonanol, 3-methylbutanal, (Z)-3-hexen-1-ol, 2-pentylfuran, linalool oxide I, and β-myrcene. Freezing–thawing and final roasting are the key processing steps for forming the aroma quality of FBT. The final roasting yielded a considerable quantity of pyrazines and pyrroles, resulting in a high-fried aroma, but caused a significant reduction in linalool, geraniol, β-myrcene, and esters, which led to a loss of floral and fruity aromas. The freezing–thawing treatment resulted in an accelerated loss of aroma substances, accompanied by a decrease in the expression level of lipoxygenase and 2-C-methyl-D-erythritol 2,4-cyclodiphosphate synthase. The formation of aroma substances in the linoleic acid metabolic pathway and terpenoid metabolic process was hindered, which had a negative impact on tea aroma. This study elucidates the causes of unsatisfactory aroma quality in tea products made from frozen tea leaves, providing theoretical support for the utilisation of frostbitten tea leaves, and helps us to understand the mechanism of aroma formation in black tea.

## 1. Introduction

Black tea is the most widely produced tea in the world, ranking second in production in China. The China Tea Marketing Association has reported that the production of Chinese black tea in 2023 reached 491,200 tons, with an output value of CNY 51.97 billion. Of this total, 379,000 tons was sold domestically [1]. Black tea is classified into three main categories: broken black tea, Congou black tea, and Souchong black tea. Congou black tea represents the most consumed category of black tea in China, characterised by a sweet and mellow taste, as well as intact leaves [2,3]. The primary processing techniques employed in the production of Congou black tea include withering, rolling, fermentation, and drying [4]. The tea cells are crushed through rolling, which gives rise to the quality characteristics of red soup and red leaves in fermentation [5]. Notable Congou black teas produced in China include Keemun black tea, Dianhong black tea, Jinjunmei black tea, Yingde black tea, Jiuquhongmei tea, and others. The quality and flavour of these teas vary according to the variety and origin [6].

Aroma is the second most important factor in the evaluation of black tea quality after taste [7]. It is a significant determinant of consumer preferences and product choices. The principal aroma types of black tea are sweet, floral, fruity, honey, fired, and smoky pine [8]. The formation of different aroma types is dependent on the specific types and proportions of the aroma components. Over 400 varieties of volatile compounds have been identified in black tea [9]. However, only a small number of these compounds have been found to exhibit aroma characteristics and are, therefore, considered to be odour-active substances [10]. Keemun black tea is considered to be among the world’s four most highly regarded black teas due to its distinctive aroma, which is known as the “Keemun aroma”. The primary aroma-active compounds responsible for this characteristic aroma are geraniol, linalool, benzeneaceetalhydrate, 2-pentylfuran, and methyl salicylate [11]. The key volatile substances responsible for the floral and caramel aromas in Dianhong black tea are identified as linalool, geraniol, phenylethanol, and 3-ethyl-2,5-dimethylpyrazine [12]. The floral and fruity aroma components of Jinmudan black tea were identified as linalool, phenylacetaldehyde, and δ-decalactone [13]. The principal components of the floral and honey-like fragrance in Hunan black tea are nonanal, nerolidol, benzyl alcohol, and phenylethanol [14].

The aroma of tea is subject to a number of influences, including the variety of tea trees, the ecological environment, and the processing technology employed [15]. However, the application of tea production technology, along with the use of different tea varieties, cultivation measures, and processing techniques, has led to a significant increase in product homogeneity. Consequently, some product developers have sought inspiration in the shaping process of green tea to develop distinctive black tea products with the aim of stimulating market consumption. They have successfully developed black tea products with a flat, needle-like, whorl-like, or granular appearance [16,17]. Researchers had previously attempted to card tea leaves into a straight shape after rolling and then flattening them to process FBT. However, the rolling process resulted in a curved and wrinkled shape, which was not sufficiently smooth and straight. FBT with a flat and straight shape has been achieved through technological innovation. The carding treatment was conducted with pressure, rather than rolling, to crush the tea cells, in conjunction with the flattening process observed in Longjing tea [18]. The additional findings indicated that freezing and thawing can effectively disrupt the structure of tea cells, accelerate the fermentation process, and enhance the colour and flavour of black tea [19]. Furthermore, this process preserves the natural shape of the buds and leaves, facilitating the formation of a flat, straight, and smooth tea shape.

In recent years, the mechanisms of aroma formation during the entire tea processing have become a subject of considerable research interest, with studies reported in six major tea categories: green tea [20,21,22], black tea [23,24,25], oolong tea [26,27], dark tea [28,29], yellow tea [30], and white tea [31]. The majority of research on the dynamic changes in black tea aroma during processing has focused on the SBT. The shaping process has been identified as a significant factor influencing the quality of black tea [32]. It may, therefore, be inferred that there are differences in aroma between FBT and SBT. Nevertheless, there is a paucity of literature examining the changes in aroma during the processing of FBT. In order to emphasise the advantages of Zhejiang Province in flat tea production, we successfully obtained FBT with a shape similar to Longjing tea and a black colour through process optimisation. The tea cells were broken by a combination of freezing and thawing with subsequent carding treatment, and the aroma was enhanced by high-temperature roasting. In this study, SBT was employed as a control, with proteomics and HS-SPME-GC-MS technology used to gain insight into the dynamic changes in aroma during the processing of FBT. This research aims to provide theoretical guidance on the formation of black tea aroma and the innovation of black tea products.

## 2. Materials and Methods

### 2.1. Chemicals and Reagents

Ethyl decanoate (99%, analytical reagent) was procured from Aladdin Biochemical Technology Co., Ltd. (Shanghai, China). n-Alkanes (C7-C30) and solid-phase microextraction (SPME) fibre (Supelco, CAR/DVB/PDMS, 50/30 μm, 2 cm) were obtained from Merck KGaA (Darmstadt, Germany).

### 2.2. Preparation of Tea Samples

**Figure 1:**
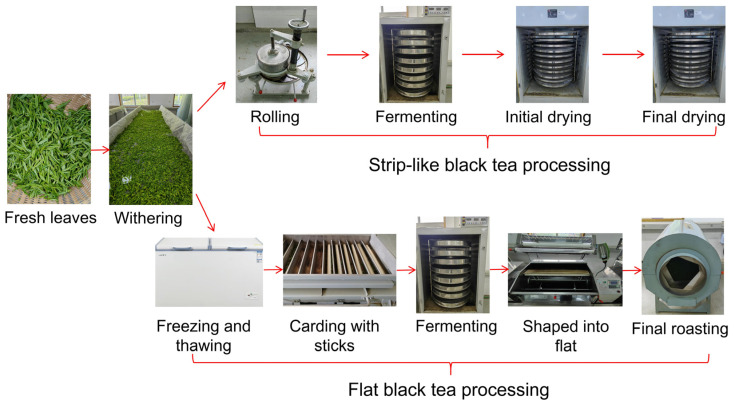
Processing flowchart of black tea.

Fresh tea leaves of the Yingshuang variety (Chinese National Approved Tea Variety, Number: GS13041-1987, one of the main tea varieties grown in Zhejiang Province) were harvested from Huanghuyunding Farm in Yuhang District, Hangzhou City, with one bud and one leaf. The tea processing flow is illustrated in Figure 1, and the specific parameters are presented in Table S1. A total of 12 tea samples were collected from each processing step, encompassing fresh leaves (FL), withered leaves (SWL and FWL), rolled leaves (SRL), fermented leaves (SFL and FFL), initial dried leaves (SIL), final dried leaves (SBT), frozen–thawed leaves (FTL), carded leaves (FCL), the leaves shaped into flat (FSL), and final roasted leaves (FBT). A total of 250 g of tea samples from each processing step (including fresh leaves) were rapidly frozen with liquid nitrogen and stored at −80 °C for the subsequent analysis. With the exception of the dried samples, all the samples were subjected to freeze-drying (at −55 °C for 48 h) and subsequently pulverised to a particle size of less than 1 mm prior to GC-MS analysis.

### 2.3. Sensory Evaluation Method

Tea infusions were prepared and subjected to sensory evaluation in accordance with the Chinese national standard (GB/T 23776-2018) [7] and the reference [33]. In summary, 3.00 g of the tea samples were brewed with 150 mL of boiling water for 5 min. The sensory evaluation of the aroma qualities of the tea soups was conducted by seven evaluators, comprising three men and four women. All the participants had undergone sensory evaluation training for a period exceeding two years. Initially, a preliminary evaluation was conducted, after which the sensory evaluation group deliberated and determined that the aroma should be evaluated according to six dimensions: overall intensity, floral, fruity, high-fired, sweet, and dull.

### 2.4. GC-MS Analysis Conditions

In total, 1.60 g of tea sample was transferred into a 100 mL sample bottle containing 80 mL of purified water and 10 μL of ethyl decanoate (0.863 g/L). Following a thorough shaking, the sample was subjected to a 10 min water bath at 50 °C to allow for equilibrium. The SPME fibre (50/30 μm DVB/CAR/PDMS) was introduced into the headspace (HS) for a period of 50 min at 50 °C. Subsequently, the fibre was maintained within the gas chromatograph injector at 250 °C for 5 min, during which time volatile desorption occurred.

An analysis of the aroma compounds was conducted using an Agilent (Santa Clara, CA, USA) 8890 gas chromatograph (GC), which was coupled to an Agilent 5977C mass spectrometer (MS). The gas chromatography column was of the HP-INNOWAX capillary type (60 m × 0.25 mm × 2.5 μm). The carrier gas was helium (99.999%), with a constant flow rate of 1.6 mL/min and a solvent delay of four minutes. The injector temperature was 250 °C. The oven temperature was regulated as follows: maintained at 50 °C for 5 min, increased at a rate of 3 °C/min to 180 °C, maintained for 1 min, increased at a rate of 10 °C/min to 240 °C, and maintained for 8 min. The ion source temperature was 230 °C under electron ionisation mode, with an electron energy of 70 eV. The scanning mode was set to full scan, with a scanning range of *m*/*z* 50–550.

### 2.5. Qualitative and Semi-Quantitative Analysis of Aroma Components

The Agilent MassHunter Workstation Software Unknowns Analysis 12.0 was used for qualitative analysis. The aroma compounds were identified by comparing the spectra with the NIST 20 mass spectral database (https://webbook.nist.gov/chemistry/ (accessed on 23 August 2024)). The retention indices were calculated according to the linear formula of the n-alkanes (C7–C30). Those with a matching rate exceeding 85% were identified as volatile substances. The concentration of each aroma substance was calculated in relation to the internal standard using the following equation: C_(i)_ = A_(i)_ × C_(s)_/A_(s)_.

C_(i)_: concentration of the aroma substances;

A_(i)_: area under the curve for the aroma substances;

C_(s)_: concentration of the internal standard;

A_(s)_: area under the curve for the internal standard.

The relative odour-active values (rOAVs) were calculated using the following equation: rOAV_(i)_ = C_(i)_/OT_(i)_. OT_(i)_ denotes the odour threshold of aroma components in water. Aroma compounds with rOAV > 1 were deemed to exert a notable influence on the aroma.

### 2.6. Proteomic Analysis Method

A 4D label-free proteomic analysis was performed on the fresh leaves, withered leaves and frozen–thawed leaves. The proteomic analysis was entrusted to Meiji Biotechnology Co., Ltd. (Shanghai, China) for testing. Protein extraction, digestion, quantification, and LC-MS/MS analysis were performed according to the literature method [34]. MS/MS spectra were searched using the MaxQuant version 2.0.3.1 software against the Camellia database. Data analysis was performed using the Majorbio Cloud platform (https://cloud.majorbio.com (accessed on 7 December 2023)). The thresholds of fold change (FC > 2 or FC < 0.5) and *p*-value < 0.05 were employed to identify differentially expressed proteins (DEPs). The DEPs were subsequently employed in GO and KEGG enrichment analyses. The functional annotation of all the identified proteins was performed using the Gene Ontology (GO) database (http://geneontology.org/ (accessed on 22 December 2023)) and the Kyoto Encyclopedia of Genes and Genomes (KEGG) pathway database (http://www.genome.jp/kegg/ (accessed on 22 December 2023)).

### 2.7. Data Analysis

All the data were expressed as mean ± SD of three replicates and significant differences were analysed by Duncan’s multiple range test using the SAS 9.3 software with a significance threshold of 0.05. The PCA and OPLS-DA of the aroma compound content were performed using SIMCA 14.1. Heat maps of aroma compounds and bubble plots of protein functional enrichment were performed using Metware Cloud, a free online data analysis platform (https://cloud.metware.cn (accessed on 13 September 2024)). Line graphs and column plots were generated using Origin 2021b.

## 3. Results and Discussion

### 3.1. Sensory Evaluation Results

**Figure 2:**
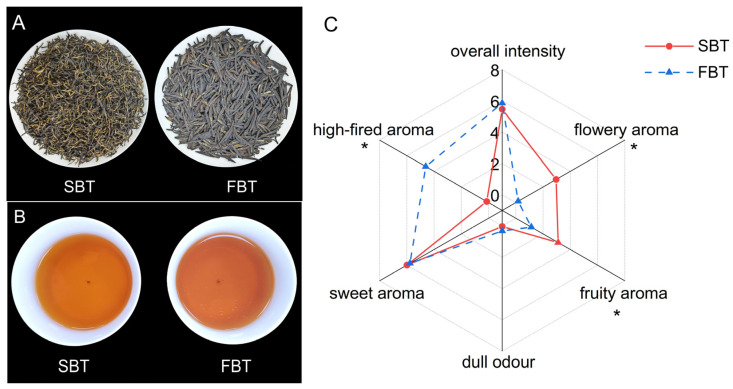
The sensory qualities of black tea. (**A**) Dried tea samples; (**B**) tea infusions; (**C**) the aroma profiles of the tea infusions. (*: *p* < 0.05).

The tea samples and infusions are shown in Figure 2. The FBT was flat, straight, and smooth in shape, similar to Longjing tea, while the SBT was curved, tightly bound, and had obvious golden hairs. The difference in appearance between the two is obvious. The infusion colour of the FBT was significantly redder than that of the other one. It can be seen that the final roasting process promoted the dissolution of internal substances, resulting in a deeper colour, which is consistent with previous research [35]. The brewed leaves of the flat black tea were intact and uniform.

The quantitative description method was used to evaluate the aroma of the tea samples. The results showed that the floral and fruity aroma intensities of the SBT were significantly higher than those of the FBT, while the high-fried aroma intensity of the FBT was significantly higher than that of the SBT. The overall intensity and sweet intensity of both were similar. It was shown that different processing methods had a significant effect on the aroma of the black tea. In order to reduce the dull odour caused by the freezing–thawing process, the FBT was finally roasted at 120–130 °C, resulting in a high-fried aroma. Long-time final drying at high temperatures is not conducive to the retention of floral aroma [36]. Meanwhile, the SBT was dried at a lower temperature, retaining more floral and fruity aroma intensity. Although we obtained an FBT which is similar in shape to Longjing tea, its aroma is not as good as that of SBT.

### 3.2. Screening of Differential Aroma Compounds

**Figure 3:**
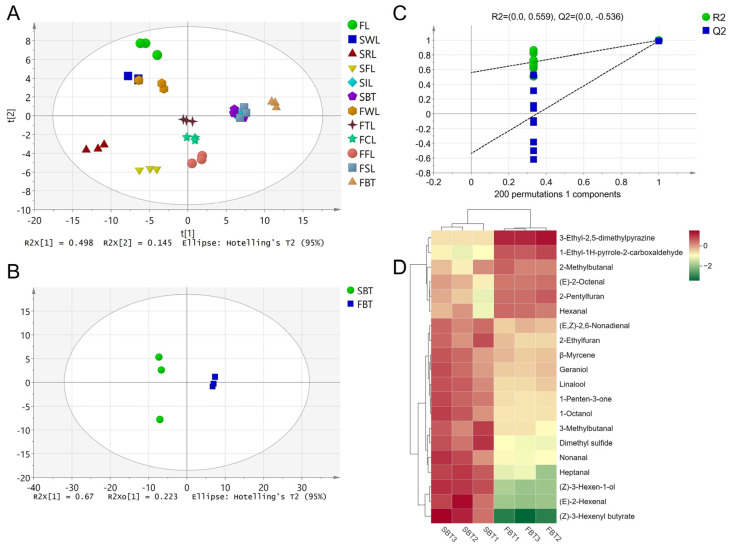
Screening of differential aroma compounds. (**A**) PCA chart of all the samples during the black tea processing; (**B**) scores of OPLS-DA (R2Y = 0.999, Q2 = 0.990); (**C**) cross-validation by 200-times permutation test (R2 = 0.599, Q2 = −0.536); (**D**) heatmap visualisation constructed with the key differential components (VIP > 1.0, OAV > 1.0, and *p* < 0.05).

By searching the MS database and comparing retention indices, a total of 92 aroma components were identified, including 25 alcohols, 18 aldehydes, 10 esters, 6 ketones, 16 heterocyclic compounds, 15 hydrocarbons, and 2 sulphur compounds (Table S2). The PCA result showed that there was a significant difference between the samples (Figure 3A). The contents of alcohols, aldehydes, esters, terpenes, aromatic hydrocarbons, and sulphur substances, and the total amount of aroma components in SBT were significantly higher than those in FBT, while the contents of pyrazines, pyrroles, and furans were in contrast. Many of the pyrazines and pyrroles exhibited a roasted aroma linked to the Maillard reaction [36,37]. On the other hand, terpenoids and esters play an important role in floral or fruity aromas [38].

An OPLS-DA analysis was performed on the aroma components between the SBT and FBT. The model achieved good discrimination results with the R2X, R2Y, and Q2 values of 0.893, 0.999, and 0.99, respectively (Figure 3B). The cross-validation results of 200 permutation tests showed that the OPLS-DA model was reliable and there was no overfitting phenomenon (R2 = 0.599, Q2 = −0.536) (Figure 3C). Among them, there were 55 aroma components with VIP values > 1. To further screen for the most important differential components, the rOAVs of the aroma components were calculated (Table S3). There were 18 components with VIP and rOAV > 1, and *p*-value < 0.05, including dimethyl sulphide, heptanal, (Z)-3-hexen-1-ol, 3-ethyl-2,5-dimethylpyrazine, 2-methylbutanal, β-myrcene, linalool, 3-methylbutanal, 1-penten-3-one, 2-pentylfuran, (E)-2-hexen-1-al, octanol, nonanal, 2-ethylfuran, geraniol, (E)-2-octen-1-al, (E,Z)-2,6-nonadien-1-al, and hexanal.

Linalool and geraniol are clearly floral components [39,40,41], with the contents in the SBT being 1.90 and 1.68 times those in the FBT, respectively. 2-Methylbutanal [42,43], 2-pentylfuran [43], 3-methylbutanal [44], octanol [45], β-myrcene [45], heptanal [46], and nonanal [46] were identified as fruity aroma compounds by GC-O. 3-Ethyl-2,5-dimethylpyrazine were identified as roasted aroma compounds [12,46]. In addition, the rOAV of 1-ethyl-1H-pyrrole-2-carboxaldehyde in the FBT was 0.75 and 2.79 times that of the SBT. It has been reported as a roasting aroma [12,47] and may contribute to the high-fired aroma. The OTs of most of the esters were high and the rOAVs were low. However, the OT of (Z)-3-hexenyl butyrate with a fruity odour was not found [48]. Its content was as high as 13.76 μg/mL and its fold change between the SBT and FBT was 12.94. These components may be the key components leading to the difference in aroma between the SBT and FBT.

Components with similar structures or aroma characters have synergistic and additive effects [49], and at low or moderate concentrations, aroma components are more likely to have additive or synergistic effects [50]. The greater the difference in chain length between similar substances, the greater the synergistic effect [51]. Esters, especially acetate esters, can enhance floral and fruity flavours through synergistic effects [52,53,54]. The ester components in this paper are mostly derivatives of hexanol, 3-hexen-1-ol, and 2-hexen-1-ol, which have similar chemical structures. It can be inferred that these substances have a synergistic effect to present fruity aroma. The aroma characteristics, aroma thresholds, and interactions of these unique ester compounds in tea are not yet very clear and are worth further research. Additionally, research has shown that (Z)-3-hexen-1-ol, linalool, and β-myrcene have a synergistic effect with fruit aroma [51]. Therefore, the high levels of these substances in the SBT may further enhance the intensity of fruit aroma.

### 3.3. Dynamic Changes in Aroma Components

The manufacturing process of the FBT was significantly different from that of the SBT. The former employed a combination of freezing–thawing and carding treatment instead of rolling to break down tea cells for the subsequent fermentation. In the drying process of the FBT, stir-frying was used, whereas in the drying process of the SBT, baking was employed. The changes in the content of different types of aroma compounds during processing are shown in Figure S1. During the processing of the SBT, the content of alcohols, aldehydes, esters, and terpenes, and the total amount of aroma substances showed a trend of first increasing and then decreasing, with the highest content observed in the SRL. The increase in terpenoids during withering and rolling is beneficial for aroma formation [25]. Furans, pyrroles, and sulphur compounds showed a gradually increasing trend. Alkanes showed a first decreasing and then increasing trend. During the processing of FBT, the content of aldehydes and esters, and the total amount of aroma compounds showed a trend of first increasing and then decreasing, with the highest content in the FWL. However, alcohols, terpenes, benzenes, and ketones showed a gradually decreasing trend. Pyrazines and pyrroles showed a gradually increasing trend. During the heat treatment process, they increased rapidly due to the Maillard reaction [55]. However, the total amount of aroma compounds rapidly decreased, which was consistent with previous studies [25].

**Figure 4:**
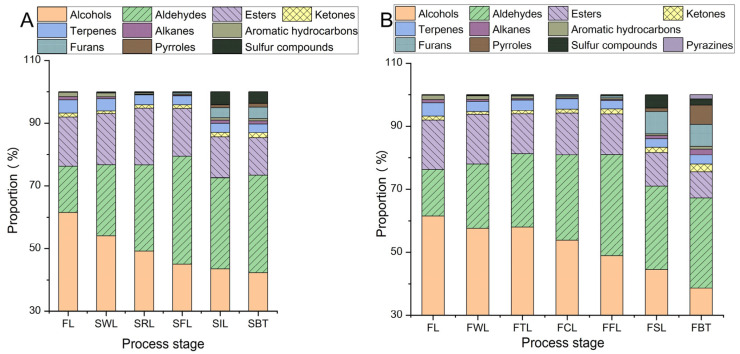
The proportion of different types of aroma substances during processing. (**A**) The manufacturing process of SBT; (**B**) the manufacturing process of FBT.

The proportions of different types of aroma compounds are shown in Figure 4. The proportions of alcohols and terpenes gradually decrease during the processing of both SBT and FBT, while the proportions of furans, pyrroles, and pyrazines gradually increase during the processing. The proportion of benzenes, alkanes, and ketones first decreases and then increases, while the proportions of aldehydes and sulphur compounds first increase and then decrease. In the SBT, the proportion of esters first increases and then decreases, with the highest proportion in the FRL. In the FBT, however, it showed a decreasing trend. Compared with the changes in aroma components between the SBT and FBT, the contents of alcohols, aldehydes, esters, ketones, and terpenes, and the total amount of aroma components decreased significantly after freezing–thawing during the FBT process. And in the later stages, aroma compounds such as alcohols, aldehydes, esters, and ketones did not increase significantly, while the rolling stage in SBT caused a significant increase in these substances. It can be seen that freezing and thawing is a key step in changing the aroma.

**Figure 5:**
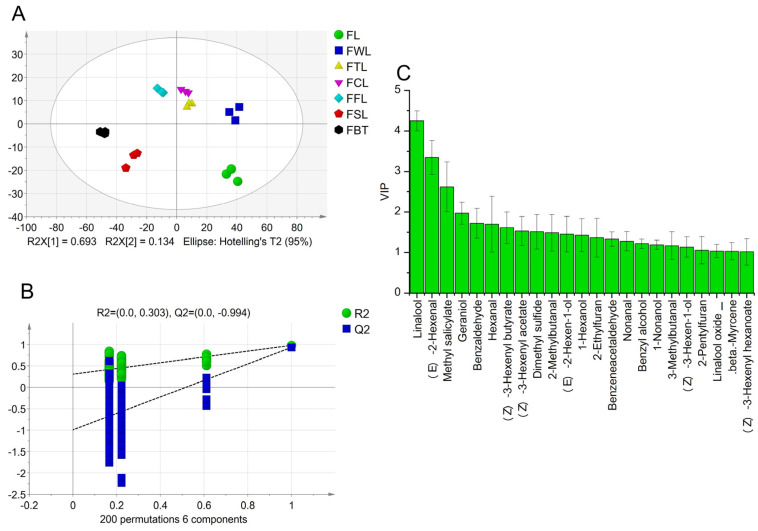
Key aroma components in FBT manufacturing process. (**A**) Scores of OPLS-DA (R2Y = 0.968, Q2 = 0.900); (**B**) cross-validation by 200-times permutation test (R2 = 0.303, Q2 = −0.994); (**C**) aroma components of variables important in projecting (VIP > 1).

The OPLS-DA analysis was used to establish a discriminant model for samples at different stages of FBT processing. The model worked well and the model was reliable with no overfitting phenomenon (Figure 5A,B). There were 23 components with VIP > 1 (Figure 5C). A total of 17 aroma components were identified as the key aroma-active compounds, excluding those with rOAV < 1, including linalool, (E)-2-hexen-1-al, methyl salicylate, geraniol, hexanal, (Z)-3-hexenyl butyrate, dimethyl sulphide, 2-methylbutanal, 2-ethylfuran, nonanal, nonanol, benzene acetaldehyde, 3-methylbutanal, (Z)-3-hexen-1-ol, 2-pentylfuran, linalool oxide I, and β-myrcene. Notably, these compounds largely corresponded to the differential components between FBT and SBT.

### 3.4. Proteomic Analysis Results

**Figure 6:**
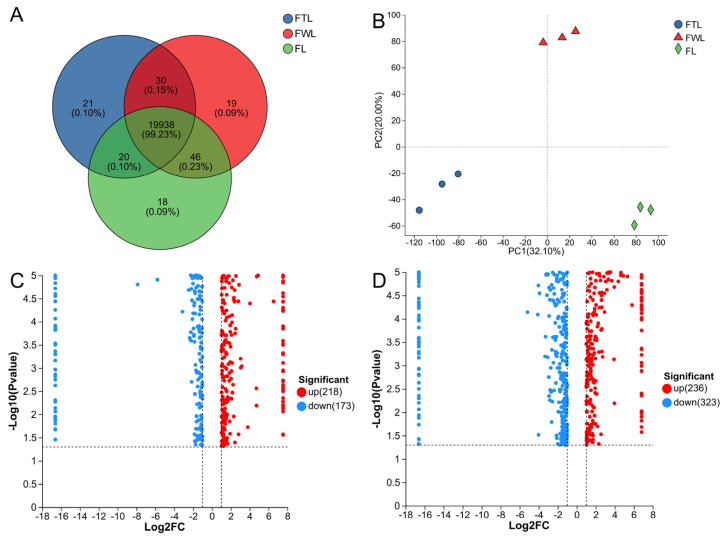
The number of proteins identified in the study. (**A**) The number of proteins identified in different sample; (**B**) PCA chat; (**C**) differential proteins as a volcano plot of FWL vs. FL; (**D**) differential proteins as a volcano plot of FTL vs. FWL.

The proteomic analysis was conducted on FL, FWL, and FTL. A total of 20,092 proteins were identified, comprising 19,938 common proteins and 58 unique proteins (Figure 6A). The PCA demonstrated that the first two principal components explained 52.1% of the original variable information, and a clear distinction was evident between the three (Figure 6B). DEPs were identified through the screening of the samples subjected to different treatments, with FC > 2 or FC < 0.5, and *p* < 0.05. The proteomic analysis revealed that the withering and freezing–thawing processes significantly impact the protein profile of the tea leaves. Specifically, FWL exhibited the upregulation of 218 proteins and downregulation of 173 proteins in comparison to FL (Figure 6C). In contrast, FTL displayed the upregulation of 236 proteins and downregulation of 323 proteins in comparison to FWL (Figure 6D).

The functional enrichment analysis was conducted on the differential proteins with a particular emphasis on the pathways associated with the metabolisms of aroma components. The GO functional enrichment analysis between the FWL and FL revealed significant differences in the biological processes, including the L-phenylalanine metabolic process, aromatic compound catabolic process, benzene-containing compound metabolic process, olefinic compound metabolic process, carboxylic acid metabolic process, carotene metabolic process, and terpene metabolic process. In the case of the upregulated proteins, significant differences were observed with regard to sesquiterpenoid biosynthetic processes, hydrolase activity (acting on glycosyl bonds), and β-glucosidase activity. The KEGG functional enrichment analysis revealed that the proteins involved in the metabolism of phenylalanine exhibited significant differences. The proteins involved in carotenoid biosynthesis, terpenoid backbone biosynthesis, fatty acid metabolism, pyruvate metabolism, glycolysis/gluconogenesis, and α-linolenic acid metabolism were found to be enriched, although no significant difference was observed (Figure S2).

The GO functional enrichment analysis between the FTL and FWL revealed significant differences in cellular component proteins, including the integral and intrinsic components of the membrane, as well as cellular anatomical entities. Additionally, there were notable distinctions in biological process proteins, particularly in the positive regulation of hydrolase activity. The changes in cellular component proteins indicated that freezing and thawing had caused significant damage to cellular structures. In the case of the upregulated proteins, a significant difference was observed in the acyl-CoA hydrolase activity proteins. The KEGG functional enrichment analysis revealed that the proteins involved in ether lipid metabolism exhibited notable differences. Diterpenoid biosynthesis, linoleic acid metabolism, terpenoid backbone biosynthesis, butanoate metabolism, carotenoid biosynthesis, citrate cycle (TCA cycle), fatty acid metabolism, and glycolysis/gluconeogenesis were found to be enriched, yet no significant difference was observed (Figure S3). The number of proteins associated with aroma metabolism during withering is significantly greater than that observed during freezing and thawing. The primary pathways for the production of volatile substances in tea are lipid degradation, terpenoid synthesis, amino acid metabolism, carotenoid degradation, and glycoside hydrolysis [3,39]. Terpenoids, phenylpropanoids/benzenoids, and fatty acid derivatives were identified as the primary volatile substances [56,57]. The aforementioned enriched pathways directly or indirectly influence the formation of aroma components.

### 3.5. Metabolic Pathway Analysis

The analysis of specific DEPs revealed that the proteins responsible for β-glucosidase activity, α-galactosidase, and a protein involved in the synthesis of methyl jasmonate in the α-linolenic acid metabolism pathway exhibited a notable increase in FWL relative to FL (Table S4). It has been demonstrated that a range of aroma precursors exist in glycoside form, and that glycosidases are capable of breaking down these precursors in order to release aromas [55,58]. The upregulation of these proteins is beneficial for enhancing the aroma during the withering process. The lipoxygenase (K00454 and K15718) and 2-C-methyl-D-erythritol 2,4-cyclodiphosphate synthase (K01770) were found to be significantly downregulated in FTL in comparison to FWL. Lipoxygenase is a pivotal enzyme in α-linolenic acid metabolism, directly influencing the synthesis of aromatic compounds such as aliphatic alcohols, aldehydes, and esters [59]. Geranyl diphosphate is a crucial component in the synthesis of terpenes, including geraniol, linalool, and myrcene, in the monoterpenoid biosynthesis pathway [3]. The downregulation of these proteins is unfavourable for the formation of aroma.

**Figure 7:**
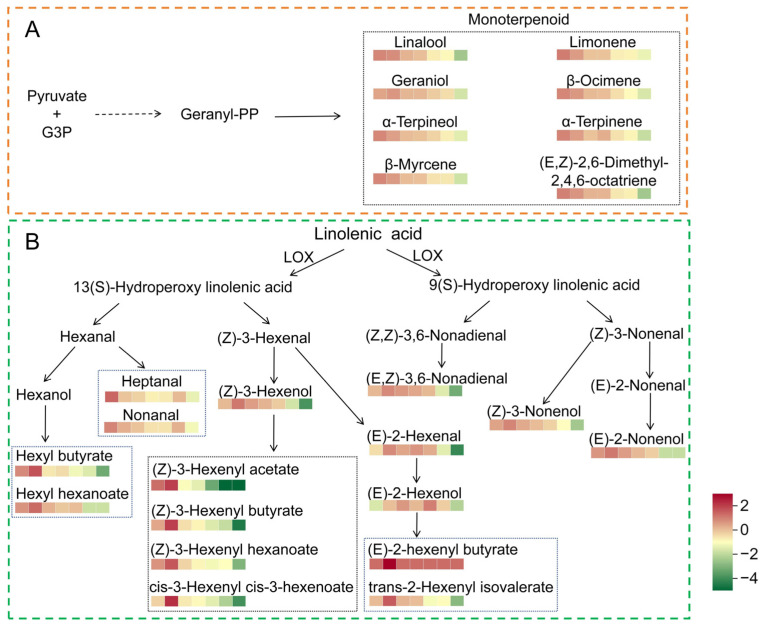
Metabolic pathway of key aroma compounds. (**A**) The synthesis pathway of terpenes; (**B**) the metabolic pathway of fatty acid-derived aroma compounds. The boxes of different colours from left to right are FL, FWL, FTL, FCL, FFL, FSL, and FBT; red and green colours represent above-mean level and below-mean level, respectively. G3P: D-glyceradehyde-3-phosphate; Geranyl-PP: geranyl diphosphate; LOX: lipoxygenase.

The metabolic pathways of the key aroma components in this study are the terpene metabolism pathway and the linoleic acid metabolism pathway. The alterations in the key aroma components associated with the regulation of these pathways during FBT processing are illustrated in Figure 7. During the withering process, the contents of (Z)-3-hexen-1-ol, (E)-2-hexen-1-al, (E)-2-hexen-1-ol and related esters, (Z)-3-nonen-1-ol, 2-nonen-1-ol, (E,Z)-3,6-nonadien-1-ol, and geraniol exhibited a marked increase. The rise in glycosidase activity is advantageous for the breakdown of glycoside precursors, resulting in an elevated geraniol content. Following the freezing and thawing process, a notable decline was observed in the contents of (E)-2-hexen-1-al, (Z)-3-hexen-1-ol and related esters, (E,Z)-3,6-nonadien-1-ol, β-myrcene, limonene, β-ocimene, α-terpinene, α-terpineol, linalool, geraniol, heptanal, nonanal, (Z)-3-nonen-1-ol, and 2-nonen-1-ol.

Among them, the concentrations of (Z)-3-hexen-1-ol, linalool, and geraniol exhibited a reduction of 27.1% to 32.2%. The concentrations of (Z)-3-hexenyl butyrate, methyl salicylate, (Z)-3-hexen-1-ol acetate, and (Z)-3-hexenyl hexanoate exhibited a notable decline, ranging from 23.0% to 86.4%. The significant decrease in content resulted in a notable weakening of the floral and fruity aroma of the FBT. It can be observed that the process of freezing and thawing not only caused cell damage and accelerated the loss of aroma compounds, but also led to a reduction in the expression of lipoxygenase and 2-C-methyl-D-erythritol 2,4-cyclodiphosphate synthase, impeding the synthesis of aroma compounds in the linoleic acid metabolism pathway and terpene synthesis pathway, particularly esters and terpenes with a floral or fruity aroma. This also elucidates the reason for the poor aroma quality of tea products manufactured from fresh tea leaves that have been frostbitten in late spring.

## 4. Conclusions

This study combined freezing–thawing and carding with sticks instead of rolling to crush tea cells to produce FBT with a shape similar to Longjing tea. The differences in aroma quality between SBT and FBT, as well as the changes in aroma components during processing were investigated. Additionally, proteomic analysis was conducted on the fresh leaves, withered leaves, and frozen–thawed leaves. The results showed that the combination of freezing–thawing and carding can achieve a good cell disruption effect, and the FBT produced has a flat and straight appearance, but it has a negative impact on the formation of floral and fruity aromas. The floral and fruity aroma of the SBT was absent in the FBT. Instead, the FBT had a pronounced high-fired aroma. The principal aroma components during the FBT process were linalool, (E)-2-hexen-1-al, methyl salicylate, geraniol, hexanal, (Z)-3-hexenyl butyrate, dimethyl sulphide, 2-methylbutanal, 2-ethylfuran, benzeneacetaldehyde, nonanal, nonanol, 3-methylbutanal, (Z)-3-hexen-1-ol, 2-pentylfuran, linalool oxide I, and β-myrcene. The key steps in the aroma formation of the FBT were freezing–thawing and final roasting. The freezing–thawing processing resulted in a reduction in the activity of lipoxygenase and 2-C-methyl-D-erythritol 2,4-cyclodiphosphate synthase, which impeded the synthesis of aroma compounds in the linoleic acid metabolism pathway and the terpenoid synthesis pathway. Additionally, it accelerated the conversion or loss of aroma substances, which is detrimental to the formation of floral and fruity aromas. The high-temperature final roasting produces pyrazines and pyrroles while causing a significant reduction in linalool, geraniol, myrcene, and esters. Therefore, the freezing–thawing and high-temperature final drying processes should be avoided when processing floral and fruity flavoured black teas.

## Data Availability

The original contributions presented in the study are included in the article/Supplementary Materials, further inquiries can be directed to the corresponding authors.

## Supplementary Materials

The following supporting information can be downloaded at https://www.mdpi.com/article/10.3390/foods13203243/s1, Figure S1: The changes in the content of different types of aroma compounds during processing; Figure S2: Bubble charts of GO and KEGG functional enrichment analysis between FWL and FL; Figure S3: Bubble charts of GO and KEGG functional enrichment analysis between FTL and FWL; Table S1: The specific parameters of black tea processing; Table S2: The content of aroma components in different tea samples; Table S3: The rOAVs of aroma compounds in SBT and FBT [60,61,62,63,64]; Table S4: Detail table of differentially expressed proteins.
