# Supplementary material for: Dynamic Changes in Aroma Compounds during Processing of Flat Black Tea: Combined GC-MS with Proteomic Analysis
Source: Foods. 2024 Oct 12;13(20):3243. doi: 10.3390/foods13203243 (PMC11507447; doi:10.3390/foods13203243)
Supplement: Supplementary file 1 [file foods-13-03243-s001.zip › Table S1.pdf]

Table S1 The specific parameters of black tea processing

Tea category	Process step	Parameters
Strip-like black tea	withering	Withered with a thickness of 2 cm~3 cm for 23 h, after first blown with hot air at 30 ℃ for 1 h.
	rolling	Rolled in a rolling machine for 1 h.
	fermenting	Fermented for 3.5 h in a fermentation machine at 35 ℃ with 90%RH.
	initial drying	Baked at 110 ℃ for 25 min, flipped once in the middle.
	final drying	Baked at 80 ℃ for 30 min after static cooling for 1 h.
Flat black tea	withering	Withered with a thickness of 3 cm~5 cm for 20 h, after first blown with hot air at 30 ℃ for 1 h.
	freezing and thawing	Frozen at -18 ℃ for 1 d, and thawed out at room temperature for 1 h.
	carding with sticks	Processed in a carding machine for 35 min to break down the mesophyll cells. During the carding process, tea leaves were hit with assortative sticks twice, each time for 10 min.
	fermenting	Fermented for 3.5 h in a fermentation machine at 35℃ with 90%RH.
	shaped into flat	Shaped in a flat tea machine at 160℃ for 7 min.The amount of leaves added each time was 120 g.
	final roasting	Carried out in a tea panning machine. After removing tea hair at room temperature for 15 min, tea leaves were roasted at 120 ℃ ~ 130 ℃ for 20 min.
