# Supplementary figures and images for: Dynamic Changes in Aroma Compounds during Processing of Flat Black Tea: Combined GC-MS with Proteomic Analysis
Source: Foods. 2024 Oct 12;13(20):3243. doi: 10.3390/foods13203243 (PMC11507447; doi:10.3390/foods13203243)

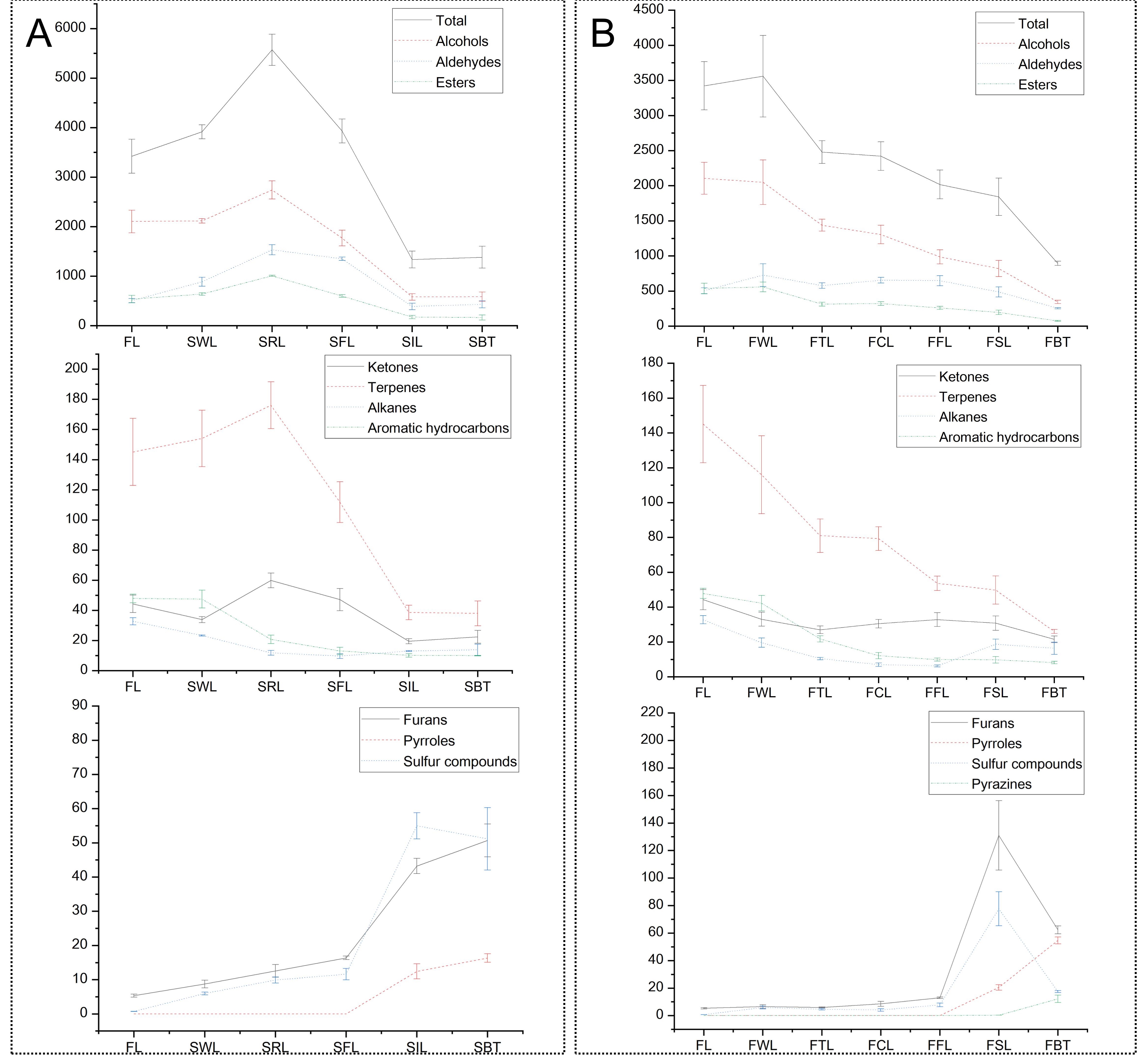

Supplement: Supplementary file 1 [file foods-13-03243-s001.zip › Figure S1.jpg]

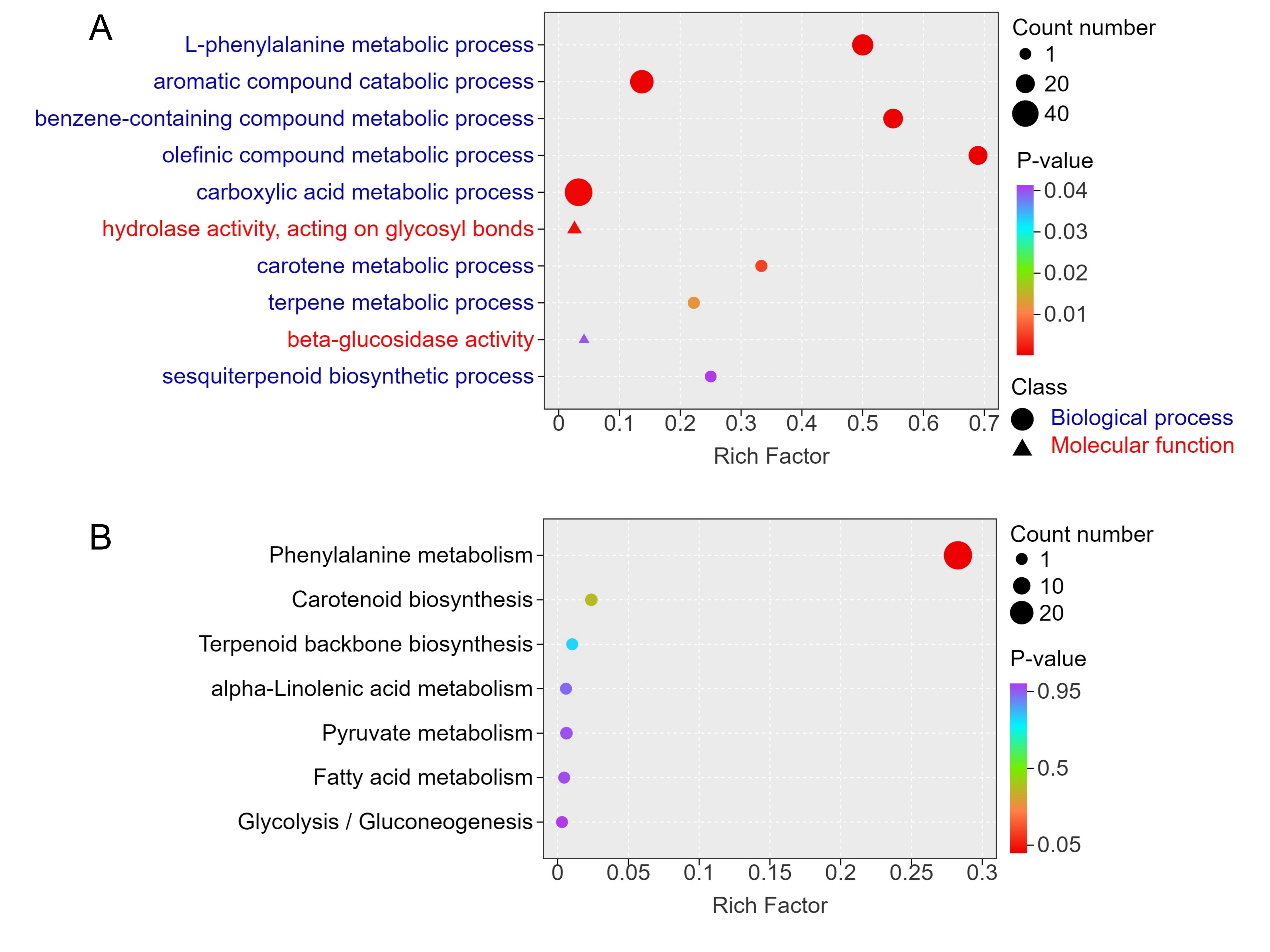

Supplement: Supplementary file 1 [file foods-13-03243-s001.zip › Figure S2.jpg]

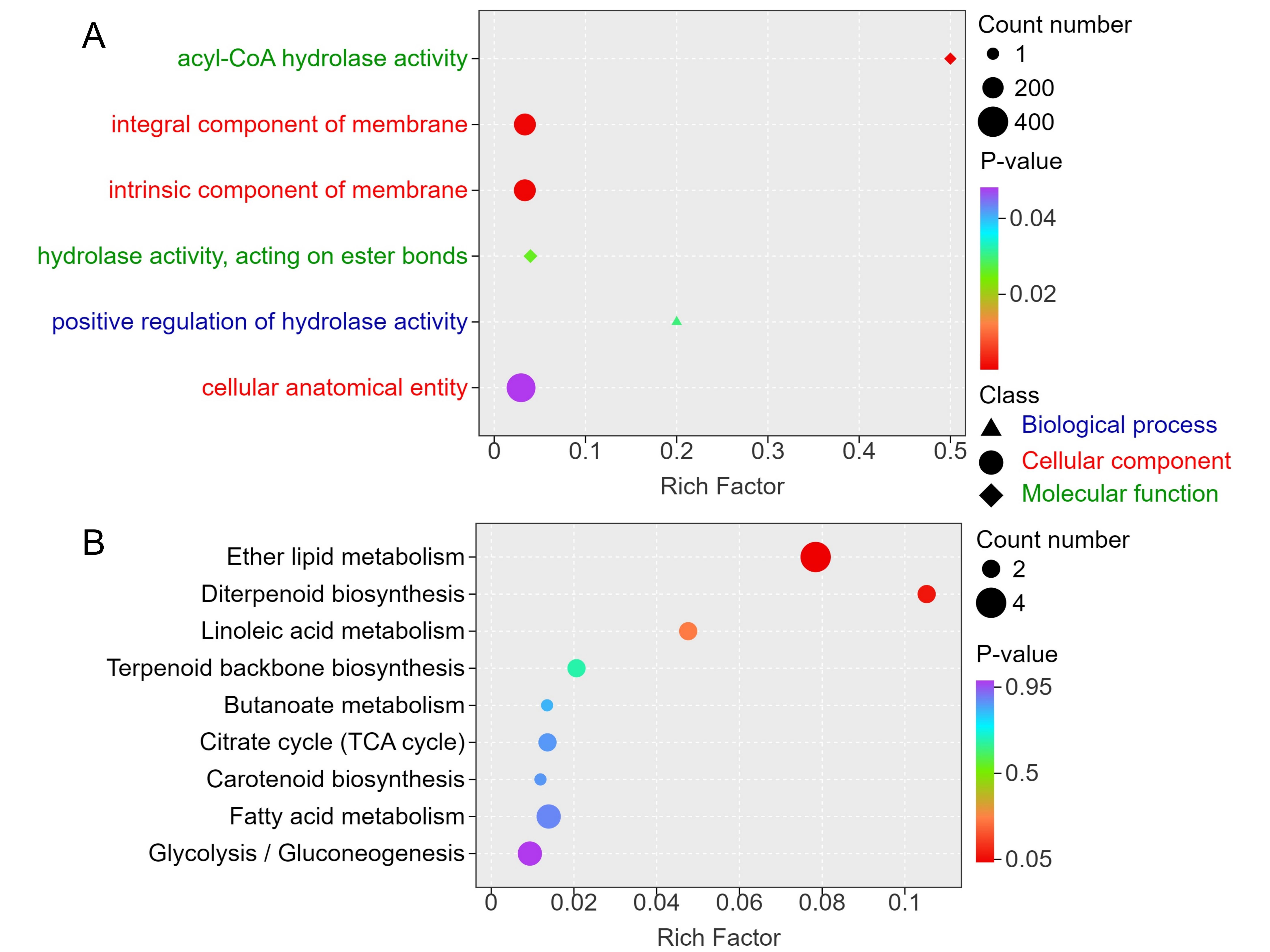

Supplement: Supplementary file 1 [file foods-13-03243-s001.zip › Figure S3.jpg]
